# Analysis of N-terminal pro-B-type natriuretic peptide and cardiac index in multiple injured patients: a prospective cohort study

**DOI:** 10.1186/cc7013

**Published:** 2008-09-12

**Authors:** Chlodwig Kirchhoff, Bernd A Leidel, Sonja Kirchhoff, Volker Braunstein, Viktoria Bogner, Uwe Kreimeier, Wolf Mutschler, Peter Biberthaler

**Affiliations:** 1 Department of Orthopedic Sports Surgery, Klinikum Rechts der Isar, Technische Universitaet Muenchen, Ismaningerstrasse 22, D-81675 Munich, Germany; 2 Department of Orthopedic Surgery and Traumatology – Campus Innenstadt, Klinikum der Ludwig-Maximilians Universitaet, Nussbaumstrasse 20, D-80336 Munich, Germany; 3 Department of Clinical Radiology – Campus Grosshadern, Klinikum der Ludwig-Maximilians Universitaet, Nussbaumstrasse 20, D-80336 Munich, Germany; 4 AO Research Institute, AO Foundation, Clavadelerstrasse 8, Ch-7270 Davos, Switzerland; 5 Department of Anesthesiology – Campus Innenstadt, Ludwig-Maximilians Universitaet, Nussbaumstrasse 20, D-80336 Munich, Germany

## Abstract

**Introduction:**

Increased serum B-type natriuretic peptide (BNP) has been identified for diagnosis and prognosis of impaired cardiac function in patients suffering from congestive heart failure, ischemic heart disease, and sepsis. However, the prognostic value of BNP in multiple injured patients developing multiple organ dysfunction syndrome (MODS) remains undetermined. Therefore, the aims of this study were to assess N-terminal pro-BNP (NT-proBNP) in multiple injured patients and to correlate the results with invasively assessed cardiac output and clinical signs of MODS.

**Methods:**

Twenty-six multiple injured patients presenting a New Injury Severity Score of greater than 16 points were included. The MODS score was calculated on admission as well as 24, 48, and 72 hours after injury. Patients were subdivided into groups: group A showed minor signs of organ dysfunction (MODS score less than or equal to 4 points) and group B suffered from major organ dysfunction (MODS score of greater than 4 points). Venous blood (5 mL) was collected after admission and 6, 12, 24, 48, and 72 hours after injury. NT-proBNP was determined using the Elecsys proBNP^® ^assay. The hemodynamic monitoring of cardiac index (CI) was performed using transpulmonary thermodilution.

**Results:**

Serum NT-proBNP levels were elevated in all 26 patients. At admission, the serum NT-proBNP values were 116 ± 21 pg/mL in group A versus 209 ± 93 pg/mL in group B. NT-proBNP was significantly lower at all subsequent time points in group A in comparison with group B (*P *< 0.001). In contrast, the CI in group A was significantly higher than in group B at all time points (*P *< 0.001). Concerning MODS score and CI at 24, 48, and 72 hours after injury, an inverse correlation was found (*r *= -0.664, *P *< 0.001). Furthermore, a correlation was found comparing MODS score and serum NT-proBNP levels (*r *= 0.75, *P *< 0.0001).

**Conclusions:**

Serum NT-proBNP levels significantly correlate with clinical signs of MODS 24 hours after multiple injury. Furthermore, a distinct correlation of serum NT-proBNP and decreased CI was found. The data of this pilot study may indicate a potential value of NT-proBNP in the diagnosis of post-traumatic cardiac impairment. However, further studies are needed to elucidate this issue.

## Introduction

Currently, severe trauma is still the leading cause of death in young patients. Whereas early post-traumatic mortality is determined by the primary traumatic impact, late mortality is caused by the development of sepsis and systemic inflammatory response syndrome (SIRS). SIRS possibly leads to multiple organ failure (MOF) and finally to multiple organ dysfunction syndrome (MODS) [1]. In this context, cardiac dysfunction in particular seems to be of striking relevance. Compelling evidence has shown a significant and independent link between inflammation and cardiac dysfunction [2]. Several cytokine signaling molecules, including endothelin-1 and Toll-like receptor, have been speculated to play important roles in the onset of cardiac dysfunction under SIRS. Involvement of these pathways in cardiac dysfunction has been convincingly validated in transgenic studies. Nevertheless, the precise mechanism of action underscoring inflammation-induced cardiac contractile dysfunction remains unclear. Regarding the assessment of cardiac function, traditional approaches include Swan-Ganz catheterization or echocardiography [3]. These techniques are either invasive or not always available under acute conditions in intensive care units (ICUs). In this context, B-type natriuretic peptide (BNP) gained significant importance in the diagnosis of congestive heart failure. BNP is a 32-amino acid protein released from cardiac ventricles in response to myocyte stretch. Although plasma levels are affected by a variety of physiological factors, BNP and especially its cleaved N-terminal pro-BNP (NT-proBNP) have been shown to be reliable serum markers for impaired cardiac function. Although there is distinct evidence that NT-proBNP might also be altered following trauma, the early dynamics of NT-proBNP in severely injured patients developing organ failure has yet to be fully characterized [4,5]. Therefore, the aims of this study were to assess the cardiac function in multiple injured patients by analyzing NT-proBNP serum levels along with an invasive hemodynamic monitoring and to correlate the results to clinical signs of MODS.

## Materials and methods

### Study design and patient collective

**Figure 1:**
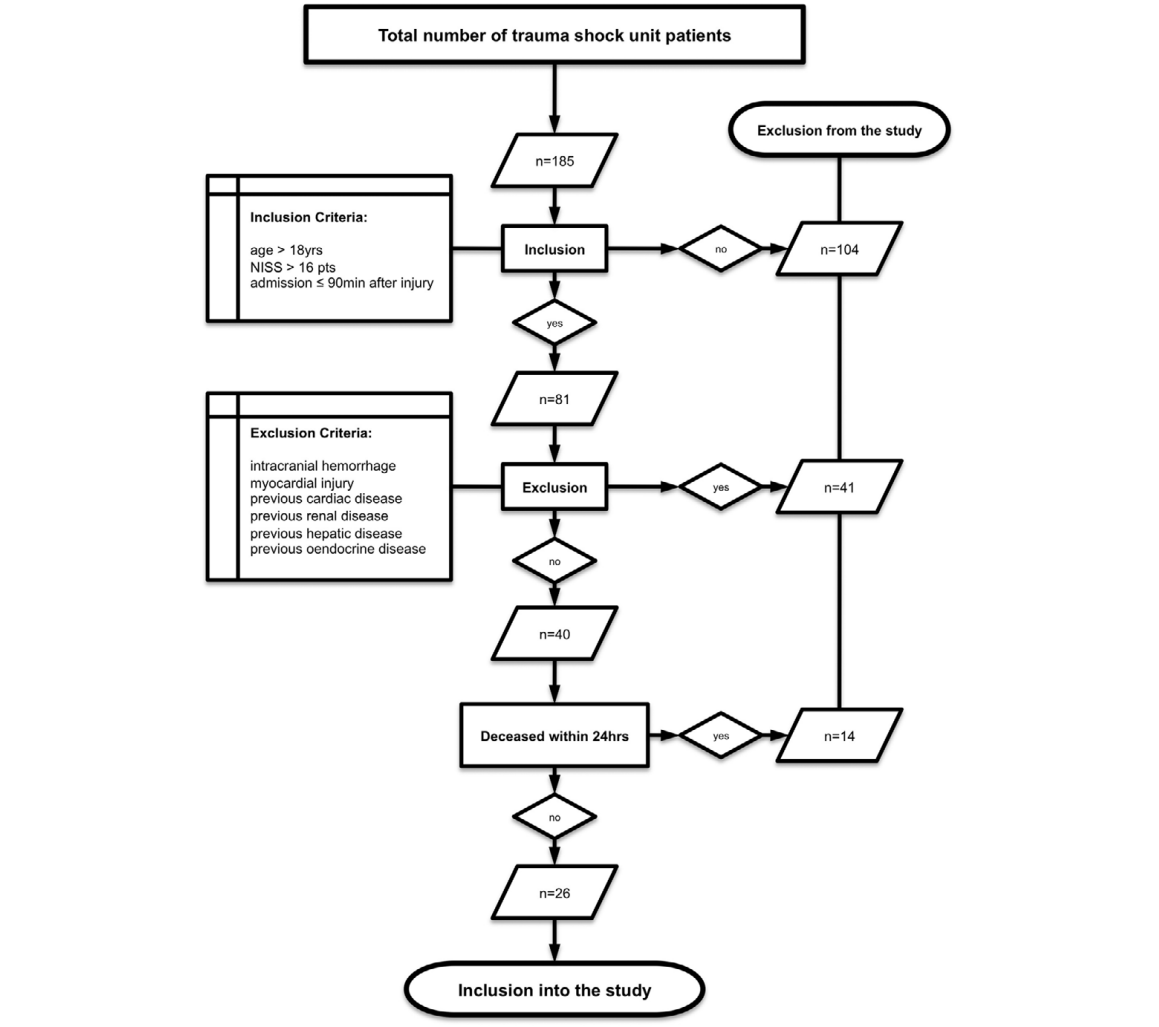
Flowchart depicting the criteria for excluding patients from the present study. The number of patients excluded for each criterion is given. NISS, New Injury Severity Score.

The study was performed between January 2005 and July 2007 at our academic level 1 trauma center according to the guidelines of Good Clinical Practice. The study was approved by the local ethics committee (reference number 012/00). Adult patients (> 18 years) arriving at the trauma shock unit within 90 minutes after trauma and suffering from multiple injury (New Injury Severity Score [NISS] of greater than 16 points) were included [6]. Written informed consent was obtained from each patient when the patient returned to consciousness or, if the patient was still unconscious, from the next of kin or a legal representative. Patients dying within 24 hours after the incident or with initial myocardial injury were excluded. Patients with traumatic brain injury, identified by signs of intracranial hemorrhage on the initial routine follow-up computed tomography scan, were excluded as well [7]. Further exclusion criteria to eliminate bias by pre-existent alterations of NT-proBNP were previous cardiac, renal, hepatic, or endocrine diseases prior to admission (for exclusion criteria, see Figure 1). After the initial resuscitation and primary surgical interventions necessary in accordance with the present standards of care, patients were admitted to the ICU. The baseline characteristics such as age, gender, mechanism of injury, and past medical history were retrieved subsequently. For the entire observation period, the 24-hour volume of fluid resuscitation as well as the 24-hour urinary output volume were recorded. All treatment data and measured parameters assessed in the trauma shock unit, operating room, and ICU were prospectively collected and recorded in a structured form database for each patient.

### Marshall multiple organ dysfunction score

The MODS score is an established and validated scoring system that includes the function of six different organ systems [8]. It combines measures of physiologic dysfunction in six components: cardiovascular (heart rate × right atrial pressure/mean arterial pressure), respiratory (arterial partial pressure of oxygen/fraction of inspired oxygen [PaO_2_/FiO_2_]), renal (serum creatinine), central nervous system (Glasgow Coma Scale score), hepatic (serum bilirubin), and hematologic (platelet count). Each component score provides a quantitative measure of physiologic function over 24 hours such that 0 represents normal function and 1, 2, 3, and 4 represent increasing physiologic derangement. Values of each component are summed on a daily basis to produce a daily score; the highest total MODS score is 24. A cumulated MODS score of greater than 4 points is associated with a 2.4-fold higher hospital mortality and a 2-fold longer ICU stay. Mortality increases from 7% to 17% and the duration of intensive care increases from 3 to 6 days [9]. MOF is defined as the occurrence of severe organ failure in two or more organ systems during the treatment period, either on the same or on different days. The MODS score was calculated on admission as well as 24, 48, and 72 hours after injury. According to the MODS score, patients were divided in two subcollectives: group A had minor organ dysfunction (cumulated MODS score of less than or equal to 4 points on 2 consecutive days) and group B had major organ dysfunction (cumulated MODS score of greater than 4 points on 2 consecutive days). The outcome of the patients was evaluated 90 days after trauma.

### Analysis of NT-proBNP in serum

According to a serial protocol, 5 mL of venous blood was collected in a sterile tube containing EDTA (ethylenediaminetetraacetic acid). Sampling points were as follows: immediately after admission to the trauma shock unit (within 90 ± 45 minutes after injury) and 6, 12, 24, 48, and 72 hours after trauma. The concentration of NT-proBNP was determined using a commercially available electrochemiluminescence immunoassay (Elecsys proBNP^® ^assay; Roche Diagnostics, Indianapolis, IN, USA), as described previously [7]. Synthetic human NT-proBNP was used for standardization.

### Hemodynamic evaluation

The hemodynamic monitoring, including permanent invasive measurement of the cardiac output (cardiac index, CI), was performed by transpulmonary thermodilution (TPTD). TPTD measurements were performed using the Pulsiocath 5-French thermistortipped catheter (Pulsion Medical Inc., Irving, TX, USA). The assessment was started on admission to the ICU and was continued for the entire observation period. The CI and extravascular lung water (EVLW) were recorded 24, 48, and 72 hours after trauma. The assessment was performed three times in a row and the values were averaged [10].

### Statistical analysis

Statistical significance between groups was determined by analysis of variance on ranks, followed by Tukey method as a *post hoc *test. A *P *value of less than 0.05 was considered to be statistically significant. For calculating the correlations between NT-proBNP values and MODS score as well as between NT-proBNP values and CI, bivariate analyses with Spearman correlation were calculated. A *P *value of less than 0.001 was considered to be statistically significant. Data are given as mean ± standard error of the mean. Analyses were performed using the Sigma Stat 3.0 software package (SPSS Inc., Chicago, IL, USA).

## Results

### Demographic and clinical data

During the study period, 40 out of 185 patients fulfilled the inclusion criteria. Fourteen patients died within 24 hours after trauma and thus were excluded. A remainder of 26 patients (20 men and 6 women) survived the observation period and were enrolled in the study (for detailed information, see Figure 1). Patient ages ranged from 23 to 75 years, with a mean of 43 ± 13 years (mean ± standard deviation [SD]), and the NISS ranged from 19 to 75 points, with a mean of 35 ± 10 (mean ± SD). Twenty-two patients made uneventful recoveries, and four with a MODS score of greater than 4 died due to MOF on days 11, 18, 34, and 46 after injury. The major reason for multiple injury was a blunt mechanism like a traffic accident or a fall from a height. The volume of fluid resuscitation required in the first 72 hours ranged from 12 to 54 L. Within-subject analysis revealed no correlation of NT-proBNP and resuscitation volume over time. Clinical baseline characteristics, such as injury patterns, age, gender, NISS, and Glasgow Coma Scale score, within the first 72 hours are given in Table 1.

**Table 1:** Clinical baseline characteristics

Patient	Injury pattern	NISS	GCS score at 0 hours	Age, years	Gender	BNP at 72 hours	MODS score at 72 hours	CI at 72 hours	Outcome at 90 days
Group A (n = 16)									

1	Lung contus. bs, spleen capsular hemat., minor nephral contus.	24	15	23	Male	225	0	4.1	Survived
2	Le Fort III°, lung contus., hemo-pneumoth. ls, bs open femur # II°	34	15	29	Male	64	0	5.7	Survived
3	Vault #, lung contus. bs, serial rib # bs, hemo-pneumoth. rs, spleen hemat.	36	13	44	Female	104	1	4.2	Survived
4	Commotio cerebri, lung contus. bs, scapula #, open fibula # II°	24	13	45	Male	105	1	4.8	Survived
5	C5 facet # with incomp. cord syndrome, lung contus. ls, liver rupture, nephral contus. ls	34	14	34	Male	24	1	4.7	Survived
6	Lung contus. ls, serial rib #, pneumoth. rs, amputation below knee, bite injury lower lip	29	14	58	Male	89	1	4.2	Survived
7	Skull base #, vault #, lung contus. bs, pneumoth. ls, serial rib # ls, radius #	29	12	36	Male	22	2	4.8	Survived
8	Serial rib # ls, pneumoth. ls, liver rupture, spleen rupture, humerus shaft #	41	13	43	Male	543	2	2.9	Survived
9	Commotio cerebri, scalp laceration, lung contus. ls, pneumoth. ls, serial rib # ls, acetabulum #, os ischiadicum #, os ilium #	45	11	32	Female	91	2	3.8	Survived
10	Lung contus. bs, serial rib # ls, spleen rupture, mesentery rupture with major blood loss, femur #	27	13	62	Male	526	2	2.9	Survived
11	Mild lung contus., amputation below knee rs, femur # rs	19	15	45	Male	158	2	4.4	Survived
12	C7 # displaced with cord contus., serial rib # ls, lung contus. bs, pneumoth. ls, spleen rupture, humerus # ls	34	14	25	Female	96	2	3.4	Survived
13	Lung contus. rs, L3 #, os ileum, os ischiadium, os pubis #, femur #, trimalleolar #	27	12	57	Female	52	3	4.1	Survived
14	Scalp contus., C5 # with complete cord syndrome, lung contus. bs, shoulder dislocation	50	13	39	Female	399	3	4.2	Survived
15	Commotio cerebri, scalp laceration, lung contus. bs, hemo-pneumoth., serial rib # ls, os ileum #, os ischiadium #, os pubis #	24	11	75	Male	364	4	3.8	Survived
16	Lung contus. bs, serial rib #, T1-3 process spinal #, femur shaft #, open tibia # III°	34	11	40	Female	276	4	4.1	Survived

Group B (n = 10)									

17	Le Fort II°, serial rib # rs, pneumoth. rs, lung contus. bs, liver rupture, multiple upper and lower extremity #	48	13	35	Male	119	5	4.1	† (18 days)
18	Lung contus. bs, L1 compression #, tibia #, bimalleolar # bs, fibula shaft #, calcaneus #	34	15	48	Male	885	5	3.3	Survived
19	Major muscle damage, humerus #, open tibia # II°, open fibula # II°	22	11	33	Male	666	5	3.2	Survived
20	Vault #, Le Fort II°, lung contus. bs, hemato-pneumoth., serial rib # ls, massive shoulder destruction	34	11	46	Male	552	5	2.8	Survived
21	Commotio cerebri, scalp laceration, lung contus. rs, lung laceration rs, hemato-pneumoth. bs, A. iliaca int. rupture rs, scapula #	41	10	34	Male	2,809	6	2.6	Survived
22	Lung contus. bs, L2 compression #, L3 L4 #, retroperiton hemat., acetabulum #, sacrum #	34	15	33	Male	2,621	7	2.6	Survived
23	Lung contus. bs, serial rib # bs, hemato-pneumoth., diaphragm rupture, mesentery laceration, femur #	57	13	59	Male	1,654	7	2.6	† (34 days)
24	Serial rib # bs, hemo. bs, sternum #, mesentery rupture, femur #	41	13	57	Male	2,813	7	3.0	Survived
25	Orbit #, thoracic cavity injury with pneumoth., aortic dissection, liver rupture < 3 segments, femur #	48	11	49	Male	3,089	11	2.1	† (46 days)
26	Serial rib #, hemo-pneumoth., lung contus. bs, amputation below knee rs, femur #	33	14	32	Male	3,785	12	2.1	† (11 days)

Group A consisted of patients with a multiple organ dysfunction syndrome (MODS) score of less than or equal to 4 points, and group B consisted of patients with a MODS score of greater than 4 points. Time values refer to time after trauma. †, deceased; #, fracture; A. iliaca int., internal iliac artery; BNP, N-terminal pro-B-type natriuretic peptide; bs, both sides; CI, cardiac index; contus., contusion; GCS, Glasgow Coma Scale; hemat., hematoma; hemato-pneumoth., hematothorax-pneumothorax; hemo-pneumoth., hemothorax-pneumothorax; hemo, hemothorax; incomp, incomplete; ls, left side; NISS, New Injury Severity Score; pneumoth., pneuomothorax; rs, right side.

### Marshall multiple organ dysfunction score

Sixteen patients (group A) had a cumulated MODS score of less than or equal to 4 points during the entire observation period. All patients within this group survived. Ten patients (group B) had a cumulated MODS score of greater than 4 points on at least 2 consecutive days; out of this group, 4 patients died. Twenty-four hours after injury, the MODS scores were 2.0 ± 0.3 points in group A versus 5.1 ± 0.4 points in group B. Forty-eight hours after trauma, the MODS scores were 1.8 ± 0.3 points in group A versus 5.9 ± 0.8 points in group B and 72 hours after injury were 1.9 ± 0.3 points in group A versus 7.0 ± 0.8 points in group B. Therefore, patients in group A had a significantly lower MODS score at all observation points (*P *< 0.001). None of the patients had a MODS score of greater than 12 points. There was no statistically significant difference between the patients with different MODS scores regarding patient age, gender distribution, or severity of injury according to NISS.

### Analysis of NT-proBNP in serum

**Figure 2:**
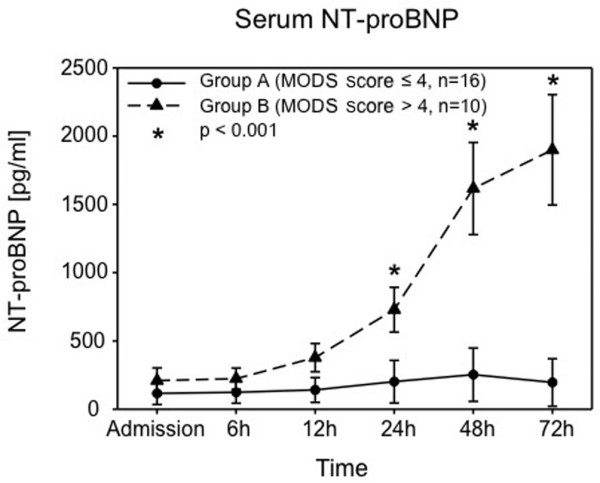
Serum concentrations of N-terminal pro-B-type natriuretic peptide (NT-proBNP) in 26 multiple injured patients. Group A (circles) consisted of patients with a multiple organ dysfunction syndrome (MODS) score of less than or equal to 4 points (n = 16 patients), and group B (triangles) consisted of patients with a MODS score of greater than 4 points (n = 10 patients). Data were calculated on admission and at 6, 12, 24, 48, and 72 hours after trauma and are presented as mean ± standard error of the mean. *P < 0.001 group A versus group B.

Serum NT-proBNP levels were increased in all 26 patients (156 samples analyzed) in comparison with norm values (*P *< 0.001). At admission, the mean serum NT-proBNP levels were 116 ± 21 pg/mL in group A versus 209 ± 93 pg/mL in group B. Six hours after injury, the serum NT-proBNP levels were 124 ± 20 pg/mL in group A versus 224 ± 78 pg/mL in group B. Twelve hours following admission, the serum NT-proBNP levels were 140 ± 23 pg/mL in group A versus 378 ± 104 pg/mL in group B. Twenty-four hours after injury, the serum NT-proBNP levels were 201 ± 39 pg/mL in group A versus 729 ± 164 pg/mL in group B. Forty-eight hours after injury, the serum NT-proBNP levels were 253 ± 39 pg/mL in group A versus 1,616 ± 337 pg/mL in group B. Seventy-two hours after injury, the serum NT-proBNP levels were 196 ± 44 pg/mL in group A versus 1,899 ± 405 pg/mL in group B. Therefore, patients in group A had lower NT-proBNP serum levels at all points of observation. This difference was statistically significant at 24, 48, and 72 hours after injury (*P *< 0.001) (Figure 2).

### Hemodynamic evaluation

**Figure 3:**
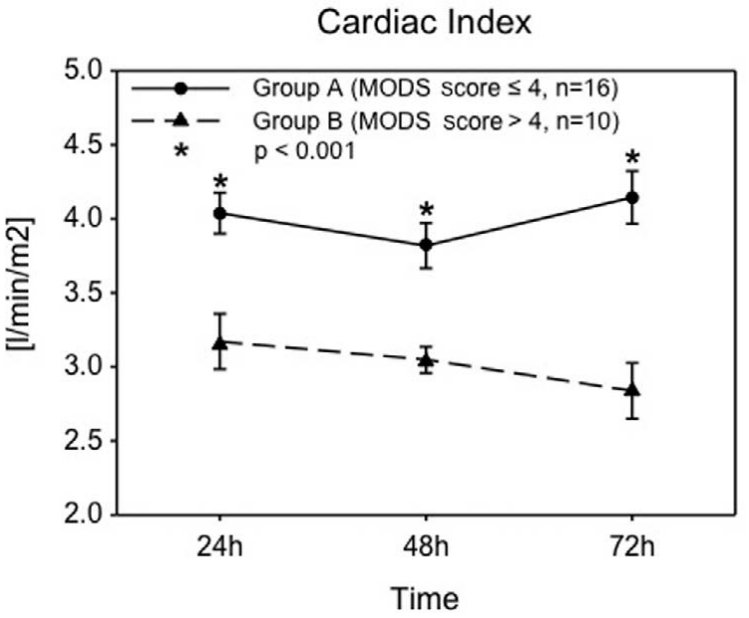
Cardiac index assessed by invasive transpulmonary thermodilution in 26 multiple injured patients. Group A (circles) consisted of patients with a multiple organ dysfunction syndrome (MODS) score of less than or equal to 4 points (n = 16 patients), and group B (triangles) consisted of patients with a MODS score of greater than 4 points (n = 10 patients). Data were calculated on admission and at 6, 12, 24, 48, and 72 hours after trauma and are presented as mean ± standard error of the mean. *P < 0.001 group A versus group B.

The CI was reduced in all patients at all time points (24, 48, and 72 hours). Twenty-four hours after injury, CIs in group A were 4.0 ± 1.4 L/minute/m^2 ^versus 3.2 ± 1.9 L/minute/m^2 ^in group B. At 48 hours, the CIs were 3.8 ± 1.5 L/minute/m^2 ^in group A versus 3.0 ± 0.9 L/minute/m^2 ^in group B. Seventy-two hours after injury, the CIs were 4.1 ± 1.8 L/minute/m^2 ^in group A versus 2.8 ± 1.9 L/minute/m^2 ^in group B. The CI of group A was significantly higher in comparison with group B at all time points (*P *< 0.001) (Figure 3). There were no statistical differences concerning EVLW observed (data not shown).

### Correlation of clinical data and NT-proBNP

A strong inverse correlation was found in comparing the cumulated MODS score and CI at 24, 48, and 72 hours after injury (*r *= -0.664, *P *< 0.001). Furthermore, there was a strong correlation in comparing the MODS score and serum NT-proBNP levels (*r *= 0.75, *P *< 0.0001).

## Discussion

In this study, we demonstrated a sequential analysis of serum NT-proBNP and simultaneously assessed cardiac output using invasive measurement. A distinct correlation of increased NT-proBNP levels and decreased cardiac output in multiple injured patients was observed 24 hours after trauma. These changes were attributed to the development of clinical signs of post-traumatic organ dysfunction.

### Diagnostic value of NT-proBNP

BNP was originally identified in extracts of porcine brain as well as in the human hypothalamus and cardiac tissue [11]. The protein is distributed as a proactive form of proBNP, comprising 108 amino acids, and is then cleaved into the biologically active BNP (32 amino acids) and an inactive 76-residue N-terminal fragment (NT-proBNP). Although only BNP turns out to be biologically active in renal target cells, the cleaved NT-proBNP can be measured with higher sensitivity and accuracy due to its longer amino acid sequence [12]. Since the NT-proBNP and BNP levels directly correspond to each other, the NT-proBNP was analyzed in this study. BNP was initially described as a biomarker for the identification of patients suffering from congestive heart failure. Elevated serum levels were also found in patients with left ventricular dysfunction and ventricular pressure overload status such as pulmonary embolism, cor pulmonale, and primary pulmonary hypertension [13].

### Post-traumatic alterations of NT-proBNP

In the present study, a significant increase of more than 100 pg/mL in NT-proBNP levels was observed on admission in all patients. Few studies have attempted to define the normal value of NT-proBNP or to find the cutoff value that allows the best balance between sensitivity and specificity. There is evidence that a single cutoff value cannot be chosen for all patients. Age, gender, body mass index, and race seem to affect the normal range of BNP. Several studies have shown that BNP values of less than 100 pg/mL are very specific for normal heart function [14]. Regarding the issue of BNP alteration in trauma patients, Kia and colleagues [15] reported BNP levels below normal as an indicator for intravascular volume loss and therefore as an initial marker of bleeding. Stewart and colleagues [4] recently analyzed BNP and transthoracic echocardiogram in trauma patients and found no correlation of BNP and cardiac dysfunction. However, there are several technical drawbacks in their study, leading to results contrary to those presented by us. In our study, invasive hemodynamic monitoring was performed using TPTD. According to the literature, thermodilution seems to be superior to transesophageal and especially transthoracic echocardiogram and currently represents the clinical standard for the determination of cardiac output [3]. We enrolled only severely injured patients with an NISS of greater than 16 points since it is well known that development of MODS significantly depends on the initial severity of injury [16]. In contrast, Stewart and colleagues [4] did not address the issue of injury severity in their work. In the present study, patients with intracranial hemorrhage were excluded in order to eliminate alteration of BNP caused by total body irradiation (TBI). This also is in contrast to Stewart and colleagues, who enrolled patients with TBI and observed an elevation of BNP independent of cardiac function. However, these findings are not new since it has been shown that systemic inflammatory reaction following TBI also leads to elevated systemic NT-proBNP levels [7]. In a second study focusing on BNP dynamics following trauma, Friese and colleagues [5] suggested that serum BNP might act as a biomarker for the preload status during resuscitation after injury. This is in contrast to our data since we did not observe significant differences regarding volume resuscitation. We also did not observe differences regarding EVLW. Friese and colleagues [5] also stated that detection of pulmonary edema on chest radiograph might not be the optimal tool to identify the presence of fluid overload. In this context, several authors demonstrated that measurement of EVLW correlates significantly to the degree of pulmonary edema and has substantial prognostic value in critically ill patients.

### Systemic inflammatory response and NT-proBNP

The main observation of this study is that patients with increased clinical signs of organ dysfunction following multiple injury had significantly increased NT-proBNP levels of greater than 200 pg/mL on admission, increasing to greater than 1,600 pg/mL 72 hours after trauma, in comparison with patients with fewer signs of organ dysfunction. Moreover, out of the group with a MODS score of greater than 4 points, four patients died. At 72 hours after trauma, these patients revealed NT-proBNP levels of up to 3,700 pg/mL. However, comparing both groups, the first significant difference regarding NT-proBNP levels was found as early as 24 hours after trauma.

Moore and colleagues [17] suggest different types or phases of MOF: an early-onset MOF (EMOF) occurring on days 0 to 3 after trauma, and a late-onset MOF (LMOF) starting 3 days after trauma. In this context, Maier and colleagues [1] differed between EMOF and LMOF with reference to the affected organ system by assessing cytokines at admission and at 24, 48, and 72 hours [1]. Although the aim of trauma research is to detect MOF as early as possible, this time protocol seems to be representative for current studies. However, we suggest a close meshed protocol as this might allow for an earlier detection of pathologic changes.

Multiple organ dysfunction in the present study was quantified according to our clinical protocol using the MODS score, first published by Marshall and colleagues [8]. The reliability of the MODS score as an outcome predictor has been demonstrated, and the correlation between a high degree of organ failure as assessed by the sequential organ failure assessment (SOFA) score and mortality is well established [8]. Peres Bota and colleagues [9] demonstrated that the MODS score and the SOFA score correlate well with the outcome in terms of mortality prediction and with the APACHE II (Acute Physiology and Chronic Health Evaluation) score. However, Ertel and colleagues [19] reported that the MODS score had a better correlation to development of SIRS and seemed to be more predictive for post-traumatic complications and outcome of injured patients.

The correlation of clinical signs of organ dysfunction and increased NT-proBNP levels is absolutely in line with other authors focusing on the predictive value of NT-proBNP in critical illness. Kandil and colleagues [20] recently confirmed the relationship between BNP level elevation and severity of sepsis independent of congestive heart failure. Also, in some patients, inflammatory cascades following cardiovascular surgery result in severe postoperative complications, including renal, hepatic, and neurological dysfunction, or respiratory and cardiovascular failure. In patients who underwent cardiac surgery, Kerbaul and colleagues [21] recently found a significant correlation of postoperative severe SIRS and elevated serum NT-proBNP concentrations. Moreover, they observed that NT-proBNP concentrations are elevated in cardiac disease in proportion to the severity of left ventricular dysfunction. The authors stated that the preoperative plasma concentrations of NT-proBNP could be a valuable predictor of severe SIRS associated with cardiovascular dysfunction [21].

Regarding the origin of BNP, Yasue and colleagues [22] determined that BNP is secreted mainly from the left ventricle in healthy adults as well as in patients with left ventricular dysfunction. They also showed that increased wall tension of the left ventricle results in an increase in the rate of BNP secretion. Because of this relationship, Yasue and colleagues proposed BNP level as a marker of the degree of left ventricular dysfunction. Although the underlying cause of SIRS-induced myocardial dysfunction remains unclear, one theory speculates on the presence of a circulating myocardial depressant substance; other investigators have shown a relationship between myocardial depression and different cytokines, including interleukin-1-beta and tumor necrosis factor-alpha [2]. These proinflammatory cytokines are known to be significantly elevated in patients with sepsis as well as in multiple injured patients [1]. The myocardial depressant effect of these cytokines has been linked to mechanisms involving nitric oxide generation [2].

## Conclusion

Twenty-four hours after trauma, serum NT-proBNP levels significantly correlate with clinical signs of MODS in multiple injured patients. Late mortality in these patients is caused mainly by multiple organ dysfunction and failure. Furthermore, serum NT-proBNP levels significantly correlate with a decreased CI as a parameter for cardiovascular function. The data of this pilot study may indicate a potential value of NT-proBNP in the diagnosis of post-traumatic cardiac impairment. However, further studies are necessary to elucidate this issue.

## Key messages

• N-terminal pro-B-type natriuretic peptide (NT-proBNP) levels are significantly increased 24 hours after severe multiple injury in patients with major signs of organ dysfunction in comparison with patients with minor organ dysfunction.

• Although this study presents only pilot data and does not allow for a direct clinical implication, NT-proBNP might serve as a tool for risk stratification in multiple injured patients.

• Further studies are necessary to analyze the value of NT-proBNP in the diagnosis of post-traumatic cardiac impairment.

## Abbreviations

BNP: B-type natriuretic peptide; CI: cardiac index; EMOF: early-onset multiple organ failure; EVLW: extravascular lung water; ICU: intensive care unit; LMOF: late-onset multiple organ failure; MODS: multiple organ dysfunction syndrome; MOF: multiple organ failure; NISS: New Injury Severity Score; NT-proBNP: N-terminal pro-B-type natriuretic peptide; SD: standard deviation; SIRS: systemic inflammatory response syndrome; SOFA: sequential organ failure assessment; TBI: total body irradiation; TPTD: transpulmonary thermodilution.

## Competing interests

The authors declare that they have no competing interests.

## Authors' contributions

CK and BAL contributed to study design and to data collection and analysis and drafted the manuscript. They contributed equally to this manuscript. SK, V Braunstein, V Bogner, UK, and WM and PB contributed to study design, data analysis, and manuscript review. All authors read and approved the final manuscript.

## References

[B1] Maier B, Lefering R, Lehnert M, Laurer HL, Steudel WI, Neugebauer EA, Marzi I (2007). Early versus late onset of multiple organ failure is associated with differing patterns of plasma cytokine biomarker expression and outcome after severe trauma. Shock.

[B2] Prabhu SD (2004). Cytokine-induced modulation of cardiac function. Circ Res.

[B3] Mathews L, Singh RK (2008). Cardiac output monitoring. Ann Card Anaesth.

[B4] Stewart D, Waxman K, Brown CA, Schuster R, Schuster L, Hvingelby EM, Kam K, Becerra S (2007). B-type natriuretic peptide levels may be elevated in the critically injured trauma patient without congestive heart failure. J Trauma.

[B5] Friese RS, Dineen S, Jennings A, Pruitt J, McBride D, Shafi S, Frankel H, Gentilello LM (2007). Serum B-type natriuretic peptide: a marker of fluid resuscitation after injury?. J Trauma.

[B6] Lavoie A, Moore L, LeSage N, Liberman M, Sampalis JS (2005). The Injury Severity Score or the New Injury Severity Score for predicting intensive care unit admission and hospital length of stay?. Injury.

[B7] Kirchhoff C, Stegmaier J, Bogner V, Buhmann S, Mussack T, Kreimeier U, Mutschler W, Biberthaler P (2006). Intrathecal and systemic concentration of NT-proBNP in patients with severe traumatic brain injury. J Neurotrauma.

[B8] Marshall JC, Cook DJ, Christou NV, Bernard GR, Sprung CL, Sibbald WJ (1995). Multiple organ dysfunction score: a reliable descriptor of a complex clinical outcome. Crit Care Med.

[B9] Peres Bota D, Melot C, Lopes Ferreira F, Nguyen Ba V, Vincent JL (2002). The Multiple Organ Dysfunction Score (MODS) versus the Sequential Organ Failure Assessment (SOFA) score in outcome prediction. Intensive Care Med.

[B10] Sakka SG, Meier-Hellmann A (1999). Cardiac output measurements. J Cardiothorac Vasc Anesth.

[B11] Saito Y, Nakao K, Itoh H, Yamada T, Mukoyama M, Arai H, Hosoda K, Shirakami G, Suga S, Minamino N (1989). Brain natriuretic peptide is a novel cardiac hormone. Biochem Biophys Res Commun.

[B12] James SK, Lindahl B, Siegbahn A, Stridsberg M, Venge P, Armstrong P, Barnathan ES, Califf R, Topol EJ, Simoons ML, Wallentin L (2003). N-terminal pro-brain natriuretic peptide and other risk markers for the separate prediction of mortality and subsequent myocardial infarction in patients with unstable coronary artery disease: a Global Utilization of Strategies To Open occluded arteries (GUSTO)-IV substudy. Circulation.

[B13] Maisel AS, Krishnaswamy P, Nowak RM, McCord J, Hollander JE, Duc P, Omland T, Storrow AB, Abraham WT, Wu AH, Clopton P, Steg PG, Westheim A, Knudsen CW, Perez A, Kazanegra R, Herrmann HC, McCullough PA, Breathing Not Properly Multinational Study Investigators (2002). Rapid measurement of B-type natriuretic peptide in the emergency diagnosis of heart failure. N Engl J Med.

[B14] Boomsma F, Meiracker AH van den (2001). Plasma A- and B-type natriuretic peptides: physiology, methodology and clinical use. Cardiovasc Res.

[B15] Kia M, Cooley A, Rimmer G, MacDonald T, Barber K, Manion P, Shapiro B, Socey J, Iddings D (2006). The efficacy of B-type natriuretic peptide for early identification of blood loss in traumatic injury. Am J Surg.

[B16] Lenz A, Franklin GA, Cheadle WG (2007). Systemic inflammation after trauma. Injury.

[B17] Moore FA, Sauaia A, Moore EE, Haenel JB, Burch JM, Lezotte DC (1996). Postinjury multiple organ failure: a bimodal phenomenon. J Trauma.

[B18] Pettila V, Pettila M, Sarna S, Voutilainen P, Takkunen O (2002). Comparison of multiple organ dysfunction scores in the prediction of hospital mortality in the critically ill. Crit Care Med.

[B19] Ertel W, Keel M, Marty D, Hoop R, Safret A, Stocker R, Trentz O (1998). [Significance of systemic inflammation in 1,278 trauma patients]. Unfallchirurg.

[B20] Kandil E, Burack J, Sawas A, Bibawy H, Schwartzman A, Zenilman ME, Bluth MH (2008). B-type natriuretic peptide: a biomarker for the diagnosis and risk stratification of patients with septic shock. Arch Surg.

[B21] Kerbaul F, Giorgi R, Oddoze C, Collart F, Guidon C, Lejeune PJ, Villacorta J, Gouin F (2004). High concentrations of N-BNP are related to non-infectious severe SIRS associated with cardiovascular dysfunction occurring after off-pump coronary artery surgery. Br J Anaesth.

[B22] Yasue H, Yoshimura M, Sumida H, Kikuta K, Kugiyama K, Jougasaki M, Ogawa H, Okumura K, Mukoyama M, Nakao K (1994). Localization and mechanism of secretion of B-type natriuretic peptide in comparison with those of A-type natriuretic peptide in normal subjects and patients with heart failure. Circulation.

